# Properties of Padding Welds Made of CuAl2 Multiwire and CuAl7 Wire in TIG Process

**DOI:** 10.3390/ma16186199

**Published:** 2023-09-13

**Authors:** Jarosław Kalabis, Aleksander Kowalski, Santina Topolska

**Affiliations:** 1 Center of Advanced Materials Technologies, Łukasiewicz Research Network—Institute of Non-Ferrous Metals, 44-100 Gliwice, Poland; 2 Department of Welding, Silesian University of Technology, 44-100 Gliwice, Poland

**Keywords:** welding wires, CuAl7, multiwire, CuAl2, HIP, corrosion

## Abstract

This paper presents the influence of the Hot Isostatic Pressing (HIP) process on the structure, mechanical properties and corrosion resistance of padding welds made using the TIG method from aluminium bronzes—CuAl7 and CuAl2 (a composite bundled wire). The tested CuAl7 material was a commercial welding wire, while the CuAl2 composite was an experimental one (a prototype of the material produced in multiwire technology). The wire contains a bundle of component materials—in this case, copper in the form of a tube and aluminium in the form of rods. The padding welds were manufactured for both the CuAl7 wire and the CuAl2 multiwire. The prepared samples were subjected to the Hot Isostatic Pressing (HIP) process, chemical composition tests were performed, and then the samples were subjected to observations using light microscopy, Vickers hardness testing, electrical conductivity tests, and apparent density determination using Archimedes’ Principle. Tribological tests (the ‘pin on disc’ method) and neutral salt spray corrosion tests were conducted. The padding weld made of CuAl2 multifiber material subjected to the HIP process is characterized by an improvement in density of 0.01 g/cm^3^; a homogenization of the hardness results across the sample was also observed. The average hardness of the sample after the HIP process decreased by about 15HV, however, the standard deviation also decreased by about 8HV. The electrical conductivity of the CuAl2 welded sample increased from 16.35 MS/m to 17.49 MS/m for the CuAl2 sample after the HIP process. As a result of this process, a visible increase in electrical conductivity was observed in the case of the wall made of the CuAl2 multiwire—an increase of 1.14 MS/m.

## 1. Introduction

Copper and aluminum alloys are the materials most commonly used in the marine industry for components operating in the corrosive seawater environment. CuAl alloys, due to their good mechanical properties, high corrosion resistance and wide availability, are most often used for the production of ship propellers, steering systems, machine parts and shafts, and in the aviation industry for engine parts. New technologies enable the production of modified construction materials, which facilitates the longer use of machines and significantly increases their reliability. The multifiber material suggested in the work is an innovative approach to the production of new welding materials, which allows for omitting the expensive melting and continuous casting processes. It is possible to make multifiber materials with a modified chemical composition based on plastic working. Such solutions are not known. After welding, the produced multi-filament material was subjected to a series of mechanical and structural tests, and pressing was performed using the HIP press. Hot isostatic pressing is a technological process used to remove the porosity of cast materials and objects produced, for example, using 3D printing techniques [1]. Additionally, the author of reference [1] notes the positive aspect of the HIP process application, which is revealed by the increase in mechanical properties and the elimination of gas porosities, e.g., in copper alloys manufactured in an unconventional way. Tungsten Inert Gas (TIG) is a welding method that is used very often due to the stability of the arc, good padding welds formation and the use of various welding materials [2,3]. This method uses a welding wire in the form of sections of about 1 m in length, which makes it easier to test new welding materials that can be manufactured in smaller series. The TIG method uses a shielding gas, which is usually compressed argon 5.0. Its role is to isolate the welded surface from the access of atmospheric air during the process. This protection guarantees the cleanliness of the padding weld, especially when welding with active metals such as copper, aluminum, and their alloys. Corrosion-resistant materials are desirable construction materials for use in many branches of industry. The group of bronzes with the addition of aluminum, which improves mechanical properties and corrosion resistance, is developing strongly, hence new applications for these types of materials are possible. So far, research on modified CuAl alloys has focused largely on improving the strength of the material by optimizing the crystal structure with the use of microalloying additives such as Ni, Zr, Cr and Ag [4,5]. The author of reference [6] describes the properties of a clad wire consisting of an aluminum core with a copper coating; this material has not been subjected to welding tests. The authors of references [7,8] study aluminum bundled in a copper pipe and describe the effect of heat treatment on the properties of copper and aluminum. The authors describe the positive aspects of heat treatment on the change in the yield strength and the influence of intermetallic compounds on the bonding of metals. Studies on new composite materials bundled in various configurations have been published; for example, the authors of references [8,9,10] describe their research on materials characterized by exceptional mechanical or electrical properties. The metals described in these publications are Cu-Nb, Cu-Ag, Cu-Fe and Cu-Al material configurations. The latter was tested by Chen et al. [11] as an alloy with low Al content in terms of tribological properties. The author describes the influence of aluminum content on the abrasion resistance of aluminum bronzes. These metals are successfully used in welding processes, and the author of reference [12] describes aluminum bronzes with additives welded, using the TIG method, onto steel. In reference [13], the author examines construction materials welded using the TIG method. The advantage of this welding method is the possibility of welding in positions convenient for the welder. Welded materials are characterized by high mechanical properties, which is demonstrated in the publication [12]. The described welding methods do not cause changes in the composition of the welded layer in relation to the electrode material (no effect of burning elements). The author of reference [14] describes the mechanisms occurring between the steel substrate and tin bronze welded using the TIG method. Bronzes are one of the most commonly used materials for welding using this method. There is a manual welding method using fluxes (A-TIG), a method described by Rana et al. in reference [15]. This method is characterized by the increased penetration of the weld material into the base material. In references [16,17,18,19,20,21,22], the authors discuss the use of welding methods in 3D printing. The presented WAAM (Wire Arc Additive Manufacturing) method is characterized by the high accuracy of the manufactured elements [17] compared to classic castings; moreover, it significantly affects the modification of the macro and microstructure [20].

## 2. Novelty of Idea

In this paper, the new concept of manufacturing materials for TIG applications was presented. There are many works which consider the conventional CuAl materials in the form of solid wires, but there is a lack of information in broadly accessible works that could be strictly connected to research on this kind of multiwire. The manufacturing of the CuAl2 multiwires is the first stage of the research on the manufacturing of the new groups of materials for TIG from the CuAl bronze group. The next step will be the production of CuAl multiwires with an increased content of aluminium, above the content currently available in the commercial welding wires—the manufacturing of multiwires provides this possibility. The idea of the work is to produce a welding wire from the broadly available semi-products in the form of pipes and rods/wires omitting the expensive casting process. It is assumed that the proposed manufacturing method of high-aluminium bronzes will be characterized by a low price, and the obtained materials will be characterized by high functional properties.

## 3. Experiment

**Figure 1:**
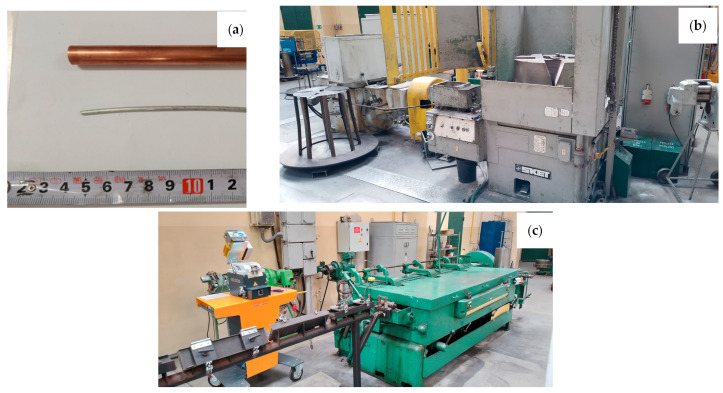
Photos of the input materials (**a**) and facilities for drawing process: SKET drum drawing machine (**b**) and wet drawing machine for drawing wires of small diameters (**c**).

The aim of the work was to determine the impact of the HIP process on the properties of padding welds manufactured using the TIG method using wires: Cu with the addition of Al 7% by weight (CuAl7) and 2% by weight (multiwire bundled three times). The CuAl7 welding wire is a material available on the welding consumables market. The multiwire with 2% aluminum is a new type of wire produced during plastic processing by drawing a bundle of materials: the matrix, which is a copper tube, and the core, which is a composite-clad wire with an aluminum core and a copper matrix. Figure 1 presents the input material for the drawing process (the copper pipe and aluminum wire), the SKET (Magdeburg, Germany) drum drawing machine (for the first stage of plastic processing) and the wet drawing machine (for drawing wires below 3 mm).

**Figure 2:**
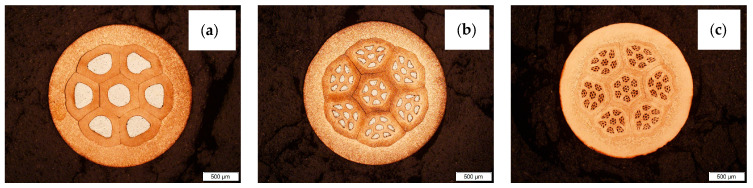
Photos of CuAl multiwires after subsequent stages of bundling: (**a**) bundling process I, (**b**) bundling process II and (**c**) bundling process III.

The multiwire concept is shown in Figure 2. Figure 2a presents the first bundle of wires (7^1^—7 aluminum fibers) which is used for the next stage, Figure 2b—the second stage of bundling (7^2^—49 aluminum fibers) and Figure 2c—the third and last stage of bundling (7^3^—343 aluminum fibers). The exponent means the number of bundling processes to which the multiwire was subjected.

The trials included the manufacturing of the CuAl multifiber wires using aluminum (Al) wires by preparing composite clad wires and their double and triple bundling into copper (Cu) pipes. The final form of the multiwires was obtained by drawing the prepared bundles to a diameter of ⌀2.15 mm. Stage I of the work on the production of multi-metal wires was the production of clad wires. A section of an aluminum wire with a diameter of ⌀3.15 mm was inserted into a copper pipe; its diameter was ⌀6.90 mm. Next, the drawing operation was performed using a chain drawbench to obtain a diameter of ⌀2.15 mm. Stage II involved cutting the obtained the CuAl clad wire into seven equal sections and bundling them into a copper tube with a diameter of ⌀9.00 mm. The bundles prepared this way were drawn to obtain a diameter of ⌀2.15 mm. The cutting and bundling operations were performed once more. The effect of the subsequent stages of bundling is shown in Figure 1. After the third stage of bundling, the multiwire was tested for fiber content, applying an automatic grain measurement method using a digital optical microscope (Keyence VHX7000, Birmingham, AL, USA) and the ratio of the surface area of aluminum fibers to the surface area of the multiwire was determined. The resulting diameter of ⌀2.15 mm is convenient for use in the TIG method. Compared to the automatic MIG/MAG method, the larger diameter of the multiwire facilitates the manual feeding of the material into the weld pool. The CuAl2 multiwires were subjected to TIG welding tests in an argon 5.0 shield. The TIG welding tests allowed for checking the quality of the produced multiwire and its functional properties. The multiwire was cut into approx. 400 mm sections. The padding welds were made on a 4 mm thick, X12Cr13 stainless steel baseplate, which enabled the controlling of the temperature of the process, so as not to allow the baseplate to bend after the welding process. As part of the work, welding tests were performed using two types of materials: the CuAl2 multiwire and the commercially available CuAl7 wire. The welding tests were conducted using a Fronius TransTig 2200 (Pettenbach, Austria) welding machine. The welding parameters are presented in Table 1. The differences in used welding parameters result from the susceptibility to welding of each particular wire and the observation of welding process stability; the multi-fiber material smoked significantly when increasing the current parameters.

Two samples of each material were cut out of the manufactured padding welds and one of them was subjected to the HIP process (AIP8-30H, Columbus, OH, USA) at a temperature of 850 °C and a pressure of 150 MPa. The content of elements in the produced padding welds was tested using a spark spectrometer (spectromaxx LMA09, Kleve, Germany). Macroscopic and microscopic observations were made using light microscopy (Olympus GX71F, Tokyo, Japan) to determine the grain size and the quality of the padding weld (cavities, microshrinkage, gasification). Vickers HV1 hardness measurements (FutureTech FM700, Kawasaki, Japan) and electrical conductivity measurements were conducted (contact device Sigmatest Foerster 2.069, Baden-Württemberg, Germany). The samples were tested for changes in density. The Archimedes Principle test was carried out using a density measuring balance by immersing the samples in ethyl alcohol. Tribological tests (Anton Paar THT, Graz, Austria) were also performed using the ‘pin on disc’ method. The tribological tests were carried out on the side wall of the padding weld after milling it. Corrosion tests were conducted in salt spray in accordance with ISO 9227 (NSS—neutral salt spray test) [23].

## 4. Results and Discussion

**Figure 3:**
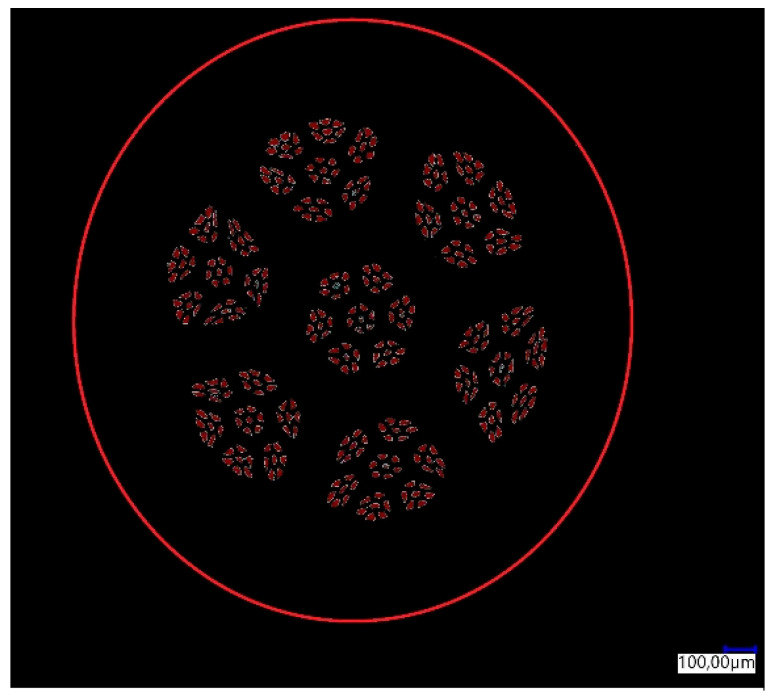
Graphical analysis of the content of aluminum fibers in the multiwire.

The percentage share of aluminum fibers in the total surface area of the sample was determined for the manufactured CuAl2 multiwire (bundled three times). The result of the analysis is shown in Figure 3. In Table 2, the results of the image analysis of the percentage content of the measurement area (aluminum fibers) in the total area (area of the cross-section of the sample) are presented.

**Figure 4:**
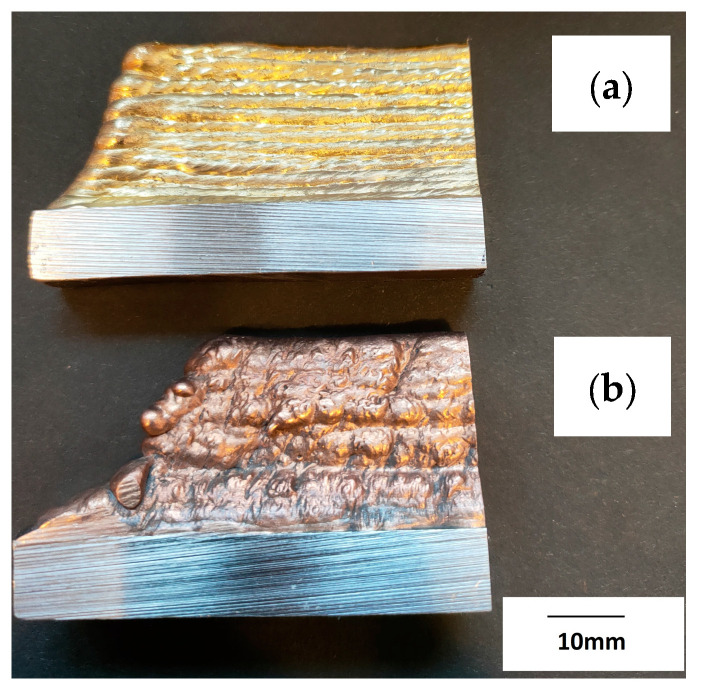
Photo of the manufactured walls: (**a**) CuAl7, (**b**) CuAl2 (bundled three times).

The prepared walls were created by the multiple padding welding of successive layers of the material (CuAl2 and CuAl7), creating several rows of padding welds with a total height of about 55 mm (Figure 4). Welding tests were conducted using the parameters listed in Table 1.

The first stage of the research was to test the chemical composition of the manufactured wall; the measurement results using a spark spectrometer are presented in Table 3.

**Figure 5:**
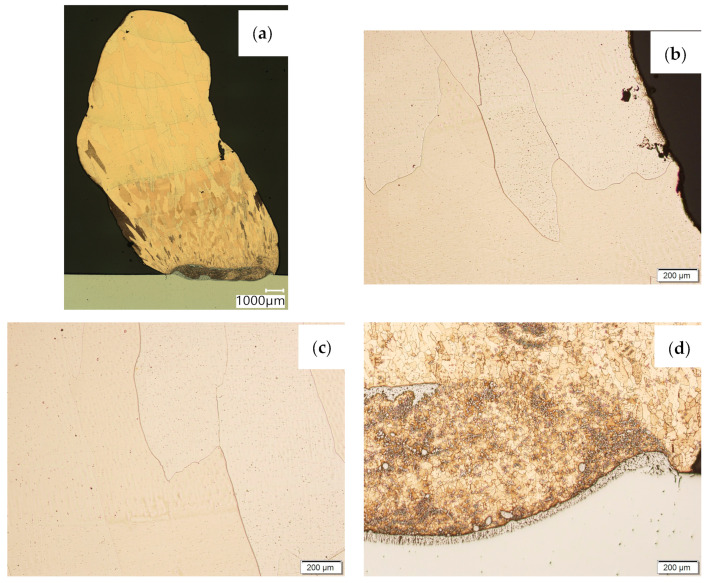
Structure of the wall manufactured using the CuAl2 multiwire: (**a**) macrostructure, (**b**) padding weld face, (**c**) padding weld middle area, (**d**) fusion area of CuAl2 with steel.

**Figure 6:**
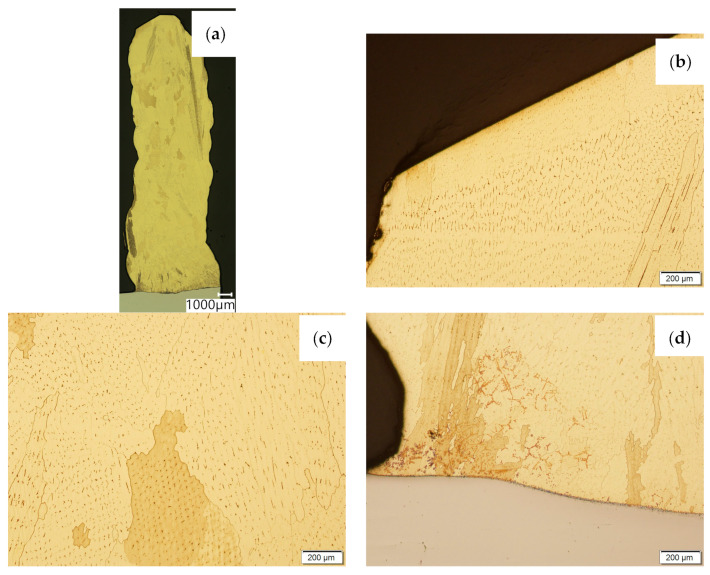
Structure of the wall made with CuAl7 wire: (**a**) macrostructure, (**b**) padding weld face, (**c**) padding weld middle area, (**d**) fusion area of CuAl7 with steel.

Then, metallographic samples (with polished and etched surfaces) were prepared for light microscope analysis. Microscopic images of the walls produced using the CuAl2 multiwire are shown in Figure 5, while those produced using the CuAl7 wire can be seen in Figure 6. In both tested materials, the surface development is characterized by a coarse-grained structure which is typical for copper alloys with the addition of aluminium (Figure 5 and Figure 6). The area at the padding weld face shows the presence of polygonal grains for both tested materials. In the case of the padding weld made with the CuAl2 multiwire, as it is closer to the area of the fusion of the alloy with the steel baseplate, a polygonal, fine-grained structure was observed (Figure 5a). Differences in the crystal structure, depending on the spot of observation, result from the way that heat dissipates during the welding of subsequent layers of the padding weld. It is important that no significant internal defects were observed inside the manufactured walls, especially for the wall made with the CuAl2 multiwire.

**Figure 7:**
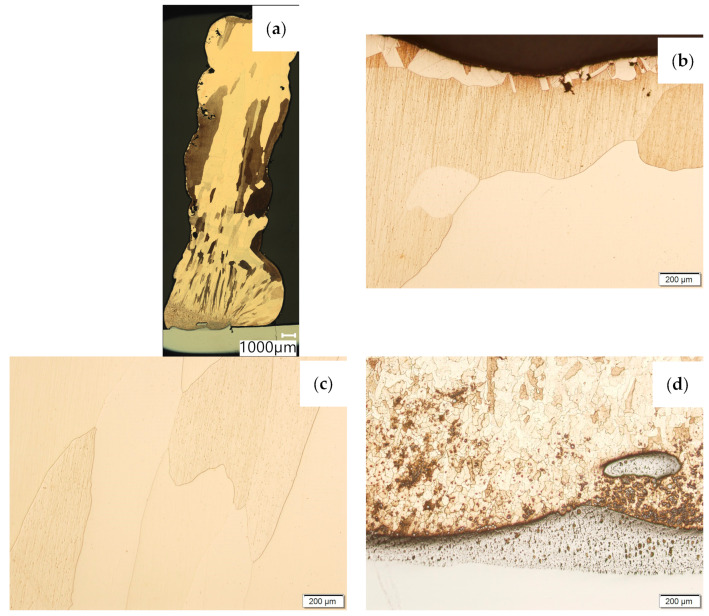
The structure of the wall produced using CuAl2 multiwire subjected to the HIP process: (**a**) macrostructure, (**b**) padding weld face, (**c**) middle area, (**d**) fusion area of CuAl2 with steel.

**Figure 8:**
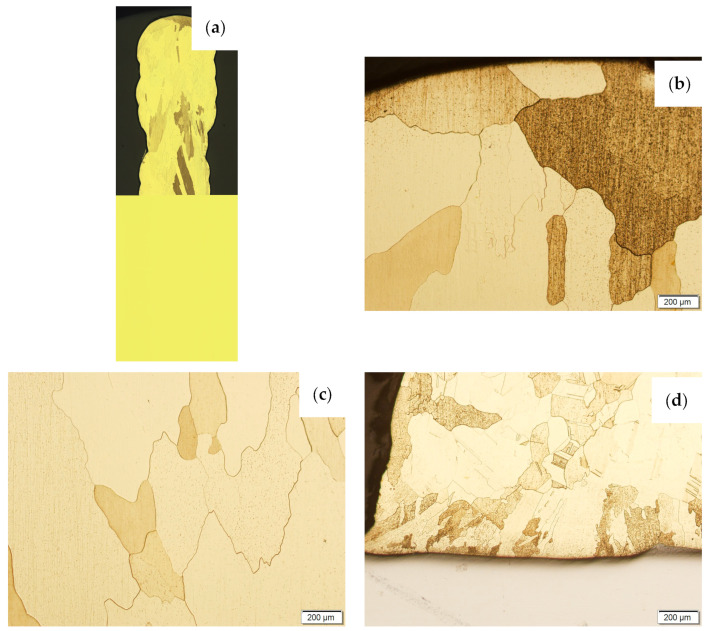
The structure of the wall made of CuAl7 wire subjected to the HIP process: (**a**) macrostructure, (**b**) padding weld face, (**c**) middle area, (**d**) fusion area of CuAl7 with steel.

Then, the samples were subjected to the Hot Isostatic Pressing process. At a temperature of 850 °C and a pressure of 150 MPa, the process of material densification applying compressed Argon 5.0 was performed. The results of the observations of the CuAl2 sample subjected to the HIP process are shown in Figure 7, and Figure 8 shows the results of the observations of the CuAl7 sample subjected to the HIP process. The microstructure of the manufactured walls in the padding weld face area after the HIP process (Figure 7 and Figure 8) is characterized by the presence of grains with clear boundaries. Nevertheless, there are visible longitudinal grains in the direction from the weld face to the area of fusion with the steel baseplate. The areas of large grains at the padding weld face were refined (Figure 7b and Figure 8b). Observations of the microstructure after the HIP process indicate the absence of defects, discontinuities or voids in the middle area of the sample.

**Figure 9:**
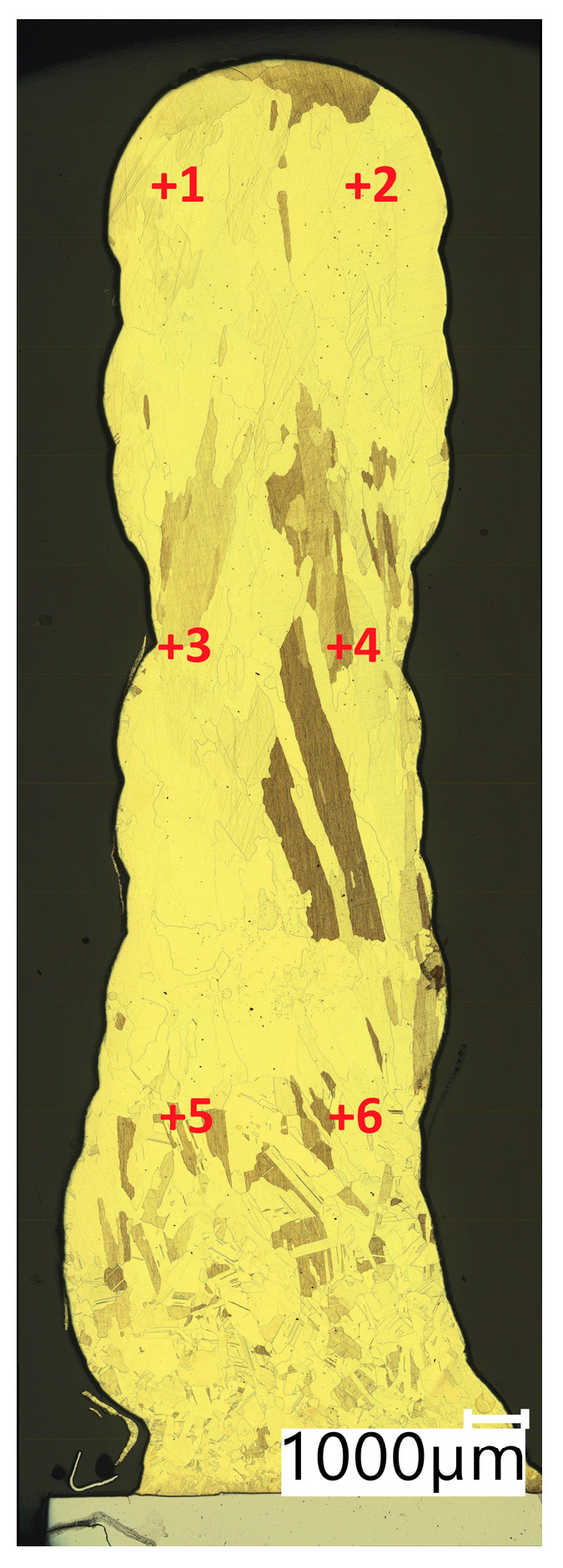
Test points of Vickers hardness test.

In Table 4, the results of Vickers hardness measurements at a load of 1 kgf are presented. The measurements were made in accordance with the diagram shown in Figure 9 (Points from +1 to +6 representing the locations where the measurement was taken).

The mean hardness value of the CuAl2 wall is 75.0HV1 with a standard deviation of 10.29HV1, while the average hardness of the CuAl2 sample after HIP processing is 59.7HV1 with a standard deviation of 2.69HV1. The hardness after the HIP process decreased, and the standard deviation also decreased significantly. The average hardness of the CuAl7 sample was 106.7HV1 with a standard deviation of 8.37HV1, while the average hardness of the CuAl7 sample after the HIP process was 75.8HV1 with a standard deviation of 8.68HV1. The HIP process contributed to the unification of the grain structure. The conductivity of the walls made using the CuAl2 multiwire is 16.35 MS/m, while after HIP it is 17.49 MS/m. Electrical conductivity tests for the walls made with the CuAl7 wire before the HIP process showed a conductivity of 7.52 MS/m, while after HIP it was 7.55 MS/m. The obtaining of clearly higher values of electrical conductivity, in the case of the wall made with the CuAl2 multiwire compared to that made with the CuAl7 wire, is due to the presence of a higher copper content in the material. As a result of the HIP process, a visible increase in electrical conductivity was observed in the case of the wall made using the CuAl2 multiwire—an increase of 1.14 MS/m.

The results of the density measurement, using Archimedes’ Principle, of both materials, before and after the Hot Isostatic Pressing process, are presented in Table 5.

The increase in density caused by the HIP application is small and, in the case of the CuAl7 sample, it was 0.02 g/cm^3^ while, for the CuAl2 sample after the HIP process, an increase of 0.01 g/cm^3^ was noted. Table 5 presents the parameters of the ‘pin-on-disc’ tribological test. In addition, before and after the process, the weights of the samples and counter-samples involved in the study were measured. The list of mass values is presented in Table 6.

Tribological tests were conducted to determine the mean value of the coefficient of friction for the friction pair: a sample made of the tested materials and a counter-sample made of 100Cr6 steel.

**Figure 10:**
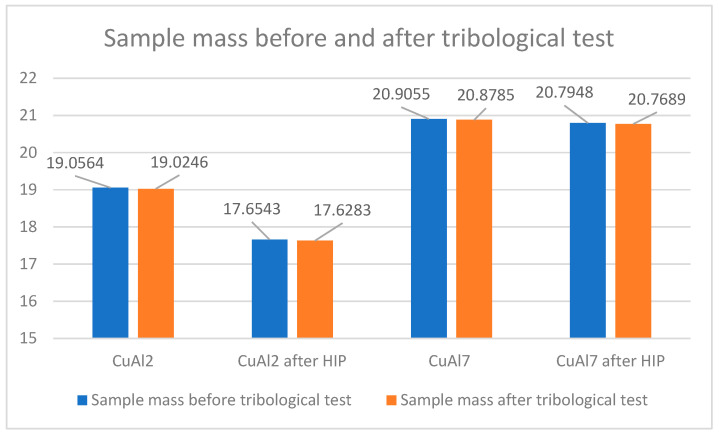
Sample weight before and after the tribological process.

Figure 10 shows the change in the weight of the samples subjected to the HIP process and, before and after the pin-on-disc process, a reduced weight loss in the CuAl2 sample after HIP can be seen while the mass of the counter-samples in the tested variants of materials does not change; therefore, these values are not included at Figure 10.

**Figure 11:**
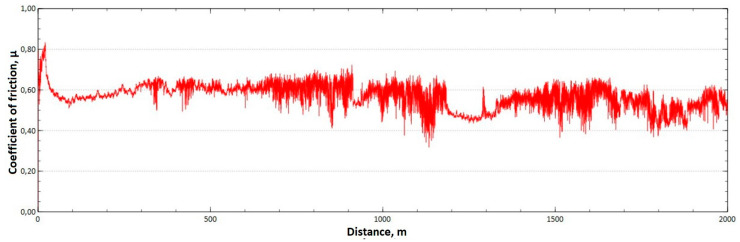
Graph of the coefficient of friction as a function of distance—CuAl2 sample.

**Figure 12:**
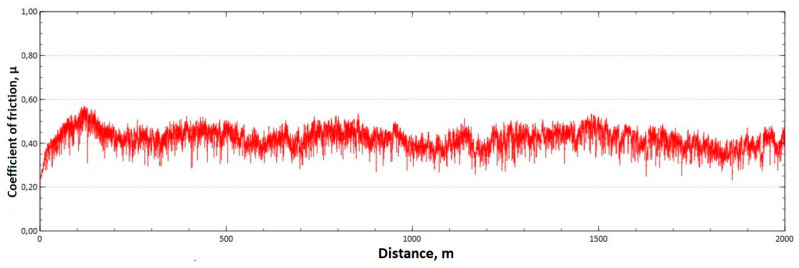
Graph of the coefficient of friction as a function of distance—CuAl7 sample.

The mean value of the coefficient of friction for the tested friction pairs was, for the samples from the CuAl2 multiwire, $\bar{µ}$ = 0.57, while for the samples from the CuAl7 wire the coefficient was $\bar{µ}$ = 0.41. Figure 11 shows the graph of the coefficient of friction as a function of the distance for the CuAl2 material, and Figure 12 for the CuAl7 material.

The analysis of the course of changes in the coefficient of friction as a function of the sliding distance for both samples after tribological tests at room temperature showed that, at the initial distance of the sliding distance, the value of the coefficient of friction increases (the so-called material grinding-in), and then, after reaching approx. 50 m for the CuAl2 sample and about 100 m for the CuAl7 sample, this value reaches a maximum, then decreases and stabilizes. Further testing in the case of the CuAl7 sample proceeded in a stable manner, with a mean value of the coefficient of friction of approx. 0.41. In the case of the CuAl2 sample, the test after the grinding-in stage was less stable and, after covering a distance of approx. 700 m, the value of the coefficient of friction fluctuates with increasing amplitude. Tribological tests on the samples after the HIP process were also performed. The aim of the study was to check the effect of the process on the value of the coefficient of dry friction. The test was conducted in accordance with the parameters in Table 6.

**Figure 13:**
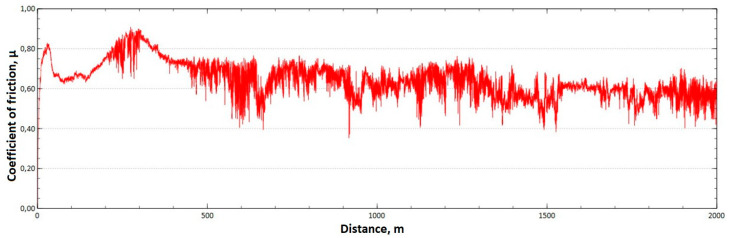
Graph of the coefficient of friction as a function of distance—CuAl2 sample after HIP.

**Figure 14:**
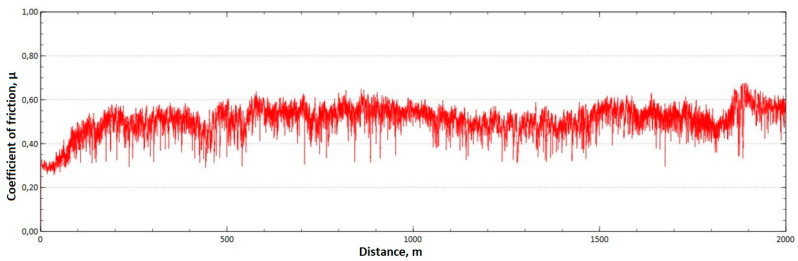
Graph of the coefficient of friction as a function of distance—CuAl7 sample after HIP.

The mean value of the coefficient of friction for the tested friction pairs was, for the samples from the CuAl2 multiwire after HIP, $\bar{µ}$ = 0.64, while for the samples from the CuAl7 wire after HIP the mean value of the coefficient $\bar{µ}$ = 0.50. In Figure 13, a graph of the coefficient of friction as a function of distance for the CuAl2 material after HIP is shown, while in Figure 14 the same graph for the CuAl7 material after HIP is presented. The analysis of the course of changes in the coefficient of friction as a function of sliding distance, for both samples after HIP after tribological tests at room temperature, showed that the overall trend of changes is similar to the test conducted for samples before HIP. At the initial distance of the sliding distance, the value of the coefficient of friction, and then, after reaching approx. 300 m for the CuAl2 sample after HIP and about 100 m for the CuAl7 sample after HIP, this value reaches a maximum, then decreases and stabilizes. Further testing in the case of the CuAl7 sample after HIP proceeded in a stable manner with a mean value of the coefficient of friction of about 0.50. In the case of the CuAl2 sample, after the grinding-in stage, the test was less stable and, after covering a distance of approx. 500 m, the value of the coefficient of friction fluctuates with increasing amplitude.

The neutral salt spray corrosion test (NSS) of samples made of the CuAl2 multiwire and the CuAl7 wire was conducted in accordance with the PN-EN ISO 9227:2017-06 standard [23]. The test parameters are listed in Table 7.

The results of the corrosion test describing the state of the solution and the process parameters for the conducted test are shown in Table 8.

**Figure 15:**
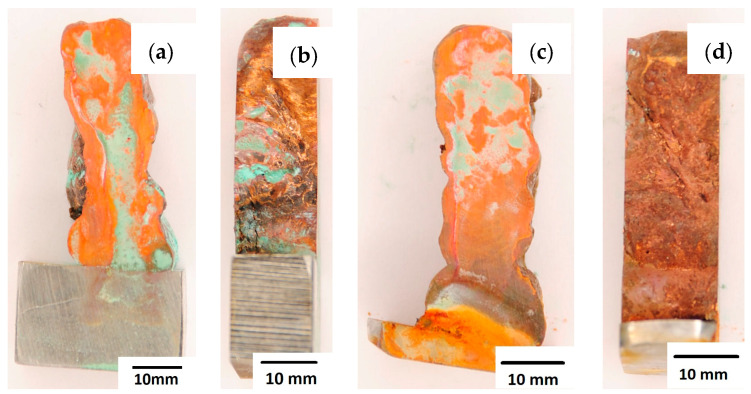
Surface of samples made with CuAl2 multiwire before the HIP process: (**a**) surface perpendicular to the direction of welding, (**b**) side surface of the wall; and after the HIP process: (**c**) surface perpendicular to the direction of welding, (**d**) side surface of the wall.

**Figure 16:**
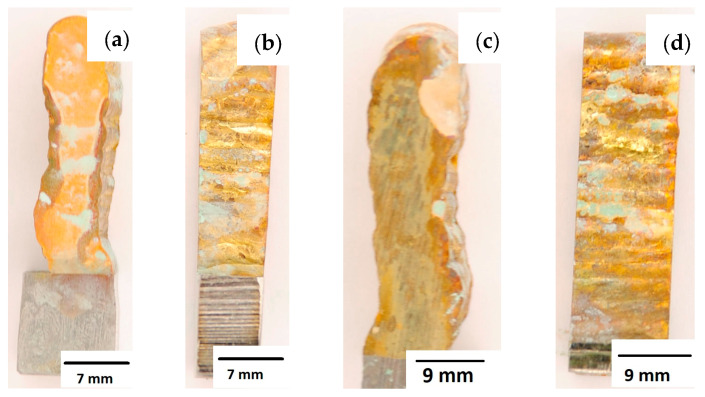
Surface of samples made using CuAl7 multiwire before the HIP process: (**a**) surface perpendicular to the direction of welding, (**b**) side surface of the wall; and after the HIP process: (**c**) surface perpendicular to the direction of welding, (**d**) side surface of the wall.

After completion of the corrosion tests, macroscopic photos of the samples were taken showing the effect of neutral salt fog on the surface of the tested materials. In Figure 15, samples made from the CuAl2 multiwire subjected to the corrosion test are shown, including (a and b) samples directly after welding and (c and d) additionally subjected to the HIP process. The sample before the HIP process (Figure 15a,b) is covered with a visible layer of corrosion products, most likely copper oxide (a typical green tint). The oxide occurs both on the surface perpendicular to the direction of welding and on the side surface of the padding weld. In the case of the samples after the HIP process (Figure 15c,d), copper oxide is present in a smaller amount on the surface perpendicular to the direction of welding, and it is concentrated in the area close to the padding weld face. In Figure 15d, the characteristic greenish tint is not visible on the side surface, which may suggest the absence of corrosion. In Figure 16, samples made from the CuAl7 multiwire subjected to the corrosion test are shown, including (a and b) samples directly after welding and (c and d) additionally subjected to the HIP process. Corrosion products were observed on the entire surface of the samples. The sample not subjected to the HIP process has less visible traces of corrosion on both tested surfaces (Figure 16a,b). The sample after the HIP process has a clear trace of green tarnish on the surface perpendicular to the direction of welding, and a green layer of corrosion products can also be seen on the side surface (Figure 16c,d).

**Figure 17:**
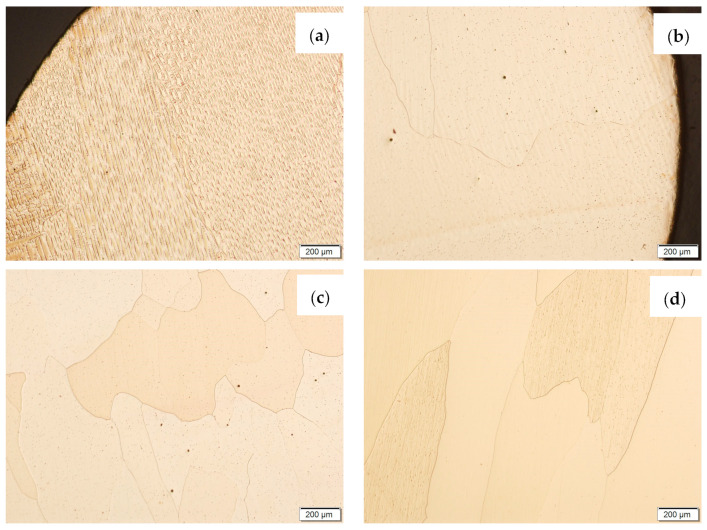
The microstructure of the wall made using CuAl2 multiwire after the NSS corrosion test before HIP: (**a**) padding weld face, (**c**) middle area of the wall; and after HIP: (**b**) padding weld face, (**d**) middle area of the wall.

**Figure 18:**
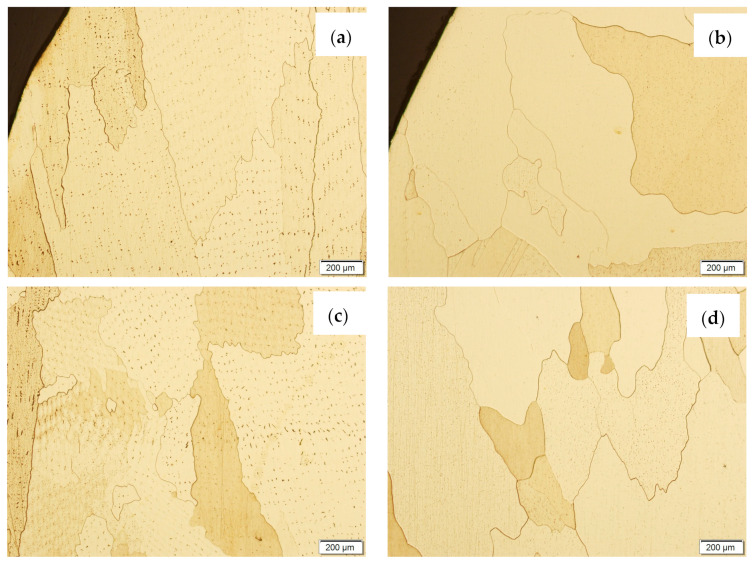
The microstructure of the wall made with CuAl7 wire after the NSS corrosion test before HIP: (**a**) padding weld face, (**c**) middle area of the wall; and after HIP: (**b**) padding weld face, (**d**) middle area of the wall.

Samples before and after the HIP process, made using the CuAl2 multiwire and the CuAl7 wire, were subjected to metallographic observations after exposure to salt fog (Figure 17 and Figure 18). The area at the padding weld face and the middle area of the manufactured wall were analysed. Figure 17 shows Na_2_Cr_2_O_7_-etched CuAl2 samples, while, in Figure 18, CuAl7 samples are presented. The wall made with the CuAl2 multiwire in the area of the padding weld face (Figure 17a) is characterized by a structure in which the effect of salt fog is clearly visible. Despite the polishing and etching necessary to make the structure visible, there are areas on the micro-section where the metal has been removed by the salt spray. The surface reveals existing pits in the area perpendicular to the direction of welding. In the middle area (Figure 17c), clear large grains of regular shape are visible; no negative effects of salt fog were observed. The wall made using CuAl2 multiwire in the area of the padding weld face after HIP (Figure 17b) is characterized by a fine-grained structure with no traces of salt fog influence on the structure. The micro-section of the CuAl2 padding weld after HIP in the middle zone also shows no traces of salt fog on the sample.

The wall made using the CuAl7 wire in the area of the padding weld face (Figure 18a) is characterized by the presence of irregularly shaped grains; there are no visible areas where the effect of salt fog was observed. In the central area of the sample not subjected to the HIP process (Figure 18c), slight losses were observed due to the action of salt fog on the surface of the sample. In the middle area of the sample subjected to the HIP process (Figure 18d), no traces of salt fog influence were observed.

## 5. Conclusions

As a result of this work, objects in the form of walls were manufactured using both the commercially available CuAl7 wire and the CuAl2 multiwire, in laboratory conditions. Based on the conducted research, the following conclusions were drawn:

1. The HIP process had a significant impact on the structure of the tested padding welds. The structure has been homogenized, and the effect of the heat-affected area in the welding process has been eliminated (homogenization of the hardness).
2. The mass loss during the tribological tests was slightly smaller for the samples subjected to the HIP process in the case of both materials.
3. The density tests using Archimedes’ Principle revealed that the CuAl2 sample showed an increase in density of 0.01g/cm^3^, while the density of the CuAl7 sample changed by 0.02g/cm^3^ after the HIP process.
4. It was found that the corrosion products were observed only on the walls’ surfaces after the test in salt fog. The samples subjected to the HIP process were less affected by the corrosion process. The microscopic images of the samples subjected to the HIP process do not show the effects of the salt fog; there are no clear traces of corrosion.

## Figures and Tables

**Table 1:** TIG welding process parameters.

Welding Process Parameters	CuAl2	CuAl7
Welding machine operating mode	2-cycle	2-cycle
Power type	AC	AC
Welding current, [A]	88	132
Arc voltage (observed range), [V]	13.0–16.5	15.8–18.0
Shielding gas, purity	Argon, 5.0	Argon, 5.0
Gas flow rate [L/min]	10	10

**Table 2:** Results of digital analysis of the percentage share of aluminum fibers in the multiwire.

Total area (Al), cm^2^	0.00178824
Number of fibers calculated, pieces	343
Graphical content Al, %	5.89
Total area (CuAl), cm^2^	0.0303675

**Table 3:** The content [%] of elements in the manufactured padding welds.

Element [%]	CuAl2	CuAl7
Cu	97.4	91.3
Al	2.01	7.72
Si	0.36	0.10
Ni	0.001	0.2

**Table 4:** HV1 hardness test results of the walls produced using the CuAl2 multiwire and the CuAl7 wire.

	No of Dents	CuAl2	CuAl2 after HIP	CuAl7	CuAl7 after HIP
face	1	85.5	56.4	115.5	83.8
	2	86.5	56.6	117.2	82.6
middle	3	75.8	59.7	102.1	84.4
	4	76.1	60.8	105.7	70.1
bottom—fusion with the baseplate	5	64.2	61.2	95.5	65.1
	6	62.0	63.2	102.7	69.0
MEAN VALUE		75.0	59.7	106.5	75.8
STANDARD DEV.		10.29	2.69	8.37	8.68

**Table 5:** Results of density measurements using the Archimedes method.

Sample	Density [g/cm^3^]
CuAl2	8.67
CuAl2 after HIP	8.68
CuAl7	7.80
CuAl7 after HIP	7.82

**Table 6:** Tribological tests parameters.

Sampling rate, Hz	60
Load, N	10
Radius, mm	8
Distance, m	2000
Linear speed, cm/s	50
Counter-sample material	100Cr6
Temperature, °C	23.6
Humidity, %	23.1

**Table 7:** NSS test parameters.

Test time, h	48.0
Time interval between checks, h	24
Chamber temperature, °C	35.0
Humidifier temperature, °C	48.0
NaCl solution, g/L	50.0
Mass loss of steel reference samples, g/m^2^	72.95

**Table 8:** Corrosion tests results of CuAl2 and CuAl7 samples.

pH of the solution	6.41
pH of the condensate	6.96
Pluviometric constant, mL/h	1.38
Condensate density, g/cm^3^	1.034
Volume of the collected solution, mL	130
Ionic conductivity of the water used to prepare the solution, µS/cm	3.68

## Data Availability

Not applicable.
